# Does aquatic exercise reduce hip and knee joint loading? *In vivo* load measurements with instrumented implants

**DOI:** 10.1371/journal.pone.0171972

**Published:** 2017-03-20

**Authors:** Ines Kutzner, Anja Richter, Katharina Gordt, Jörn Dymke, Philipp Damm, Georg N. Duda, Reiner Günzl, Georg Bergmann

**Affiliations:** 1 Julius Wolff Institute, Charité - Universitätsmedizin Berlin, Berlin, Germany; 2 Olympiastützpunkt Berlin, Berlin, Germany; University of Memphis, UNITED STATES

## Abstract

Aquatic exercises are widely used for rehabilitation or preventive therapies in order to enable mobilization and muscle strengthening while minimizing joint loading of the lower limb. The load reducing effect of water due to buoyancy is a main advantage compared to exercises on land. However, also drag forces have to be considered that act opposite to the relative motion of the body segments and require higher muscle activity. Due to these opposing effects on joint loading, the load-reducing effect during aquatic exercises remains unknown. The aim of this study was to quantify the joint loads during various aquatic exercises and to determine the load reducing effect of water. Instrumented knee and hip implants with telemetric data transfer were used to measure the resultant joint contact forces in 12 elderly subjects (6x hip, 6x knee) *in vivo*. Different dynamic, weight-bearing and non-weight-bearing activities were performed by the subjects on land and in chest-high water. Non-weight-bearing hip and knee flexion/extension was performed at different velocities and with additional Aquafins. Joint forces during aquatic exercises ranged between 32 and 396% body weight (BW). Highest forces occurred during dynamic activities, followed by weight-bearing and slow non-weight-bearing activities. Compared to the same activities on land, joint forces were reduced by 36–55% in water with absolute reductions being greater than 100%BW during weight-bearing and dynamic activities. During non-weight-bearing activities, high movement velocities and additional Aquafins increased the joint forces by up to 59% and resulted in joint forces of up to 301%BW. This study confirms the load reducing effect of water during weight-bearing and dynamic exercises. Nevertheless, high drag forces result in increased joint contact forces and indicate greater muscle activity. By the choice of activity, movement velocity and additional resistive devices joint forces can be modulated individually in the course of rehabilitation or preventive therapies.

## Introduction

Aquatic exercises are widely used in rehabilitation or preventive therapies in order to enable mobilization and muscle strengthening. Due to the buoyant force of the water a load reduction of the musculoskeletal system is expected, while drag force of the water can be modulated by the movement velocity and surface area.

For patients with osteoarthritis aquatic exercises have been strongly recommended as nonpharmacologic therapy [1]. Studies demonstrated improved physical function, strength, and quality of life after aquatic physical therapy [2–4]. Also patients with other musculoskeletal conditions [5, 6] and healthy elderly people [7] can benefit from aquatic exercise. Furthermore aquatic exercises have been recommended following total joint replacement [8, 9] with a positive effect on mobility, muscle strength, and cross-sectional area [9, 10]. Evidence about an advantage of aquatic exercises over a comparable training program on land with regard to pain relief [11] or function and mobility [12] does not exist.

As a clear benefit of aquatic exercises the load reducing effect of water is generally cited. The apparent body weight (BW) in water (F_a_) is defined as the gravitational force (F_g_) minus the buoyancy force (F_b_) and it has been shown that F_a_ decreases to about 30%BW in chest-high water [13]. However, during movement in water drag forces act opposite to the relative motion of the body segments. For overcoming those drag forces higher muscle activity is required which in turn lead to increased joint loads.

Attempts have been made to determine joint loads in water using ground reaction forces, analytical models and electromyography [14, 15]. In comparison to walking on land, peak vertical ground reaction forces were reduced in water by 63 to 70% [16–18]. During stationary running a 45% reduction of vertical ground reaction force was measured [19]. Calculations using inverse dynamics indicate joint force reductions of 65% (knee joint) and 62% (hip joint) when walking in chest-high water [13]. However, due to the complexity of hydrodynamics and the difficult determination of muscle forces (including co-contraction), the calculation of joint loads remains challenging.

The aim of this study was to quantify the load reducing effect of water during aquatic exercise and to determine the influence of increased water resistance on hip and knee joint loading. Therefore, joint contact forces were measured *in vivo* during various aquatic exercises in a group of patients with instrumented hip and knee implants.

## Materials and methods

### Instrumented implants

Instrumented knee and hip implants with telemetric data transmission were used to measure joint contact forces in vivo. The instrumented knee implant [20] is based on the Innex FIXUC system (Zimmer GmbH, Winterthur, Switzerland), a cruciate sacrificing design with an ultracongruent tibial inlay. The tibial component was modified and equipped with six strain gauges to measure the load-dependent strains in the implant. The instrumented hip implant [21] is based on the CTW prosthesis (Merete Medical GmbH, Berlin, Germany) a ‘Spotorno’ design. Inside the hollow neck, six strain gauges are applied to measure the deformation of the neck. In both implants the signals are sensed and transmitted by an inductively powered telemetry circuit [22]. After calibration of each implant 3 force and 3 moment components acting on the femoral head or the tibial component are determined from the measured strains at a sampling rate of about 100 Hz. In this study only the resultant joint contact forces (F_res_) are being analyzed and stated in percent of the subject’s body weight (%BW).

To allow safe underwater usage, the external measurement system was modified and approved by the regulatory authorities. For the power supply an induction coil with a protective grounding conductor was placed around the thigh or shank of the subjects. An external antenna was placed at the thigh or shank of the subjects to receive the signals of the implants. Both, induction coil and the external antenna were completely sealed to enable the safe underwater usage. In order to avoid movement of the power coil and antenna in water due to drag forces, a neoprene cuff and additional straps were used for fixation. Tests in water revealed that the necessary power supply of 5 mW for the telemetry unit [20] was provided and excellent signal transmission to the attached antenna was confirmed.

### Subjects

Twelve subjects with instrumented implants (6 x knee, 6 x hip) participated in this study (Table 1). The group of TKR and THR subjects were comparable with regard to body weight and height. The median age differed by 15 years with THR subjects being considerably younger than TKR subjects. Measurements were taken 2.3–8.1 years post-operatively while all subjects were free of pain. The study was approved by the ethics committee of the Charité –Universitätsmedizin Berlin. All subjects provided written informed consent to the procedures.

**Table 1:** Subject data. Median (range).

	TKR subjects	THR subjects
Sex [m/f]	2 female, 4 male	2 female, 4 male
Age [years]	77 (66–80)	62 (55–71)
Weight [kg]	90 (63–106)	90 (84–98)
Height [cm]	173 (166–175)	174 (162–179)
Years post-op	6.8 (6.2–8.1)	3.5 (2.3–4.7)

### Experiment

The aquatic exercises were performed at a swimming pool of the Olympiastützpunkt Berlin with a water height of 1.24 m. In order to adjust the water height to chest-level (approximately Xiphoid process) additional platforms were used. A lateral window in the pool allowed to track and video-tape the movements of each subject. Exercises on land were performed on the same day prior to the aquatic ones.

The exercises were divided into three groups: (A) non-weight-bearing, (B) weight-bearing and (C) dynamic activities (Table 2). All non-weight-bearing activities in water and on land were performed at a ‘slow’ frequency of 35 bpm. In order to achieve this frequency acoustic feedback with a metronome was provided.

**Table 2:** Exercises.

Group	Exercise	Description
(A) Non-weight-bearing exercises	cycling	on land: in sitting position with legs lifted (no ergometer) in water: in supine position, supported by a floating device (pool noodle)
	flexion/-extension	in standing position (on contralateral leg) TKR subjects: ipsilateral knee flexion (max. flexion)—extension (0°) THR subjects: ipsilateral hip flexion (approx. 45°)—extension (0°), with straight knee
	hip abduction/-adduction	in standing position (on contralateral leg) ipsilateral hip abduction (approx. 30°)—adduction (0°), with straight knees
(B) Weight-bearing exercises	one-legged stance	standing on the ipsilateral leg
	walking	level walking at self-selected speed
	knee bend	self-selected knee flexion angle, head above water level
	stair ascending	stair height 18 cm
	stair descending	stair height 18 cm
(C) Dynamic exercises	high-knee running	stationary running with exaggerated knee lifts
	heel-to-butt running	stationary running with bringing the heels to the butt
	jumping jack	jumping to a position with legs spread out (hip abduction) and back (hip adduction), hands on waist
	jumping lunge	jumping to a position with legs spread out (hip flexion/extension) and back, hands on waist

To investigate the influence of the movement velocity, knee flexion/extension (TKR subjects) and hip flexion/extension (THR subjects) were additionally executed at a ‘fast’ velocity of 70 bpm and ‘very fast’, i.e. as fast as individually possible, without acoustic feedback.

**Fig 1:**
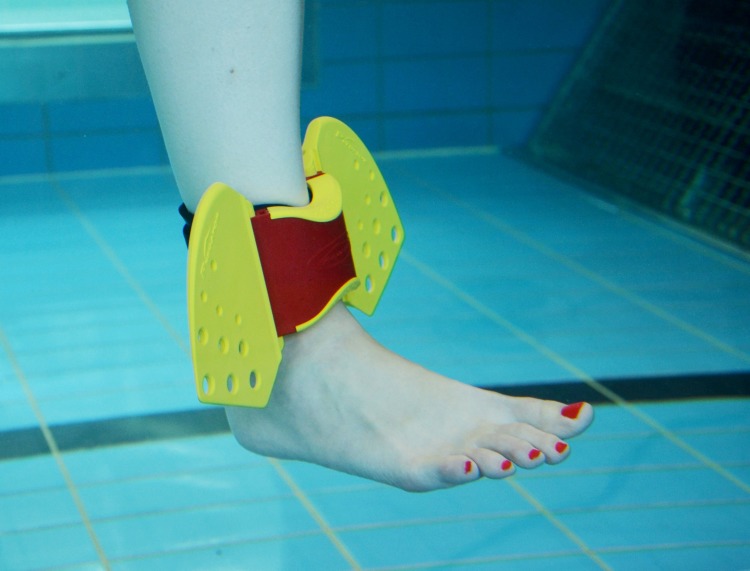
Aquafins.

For investigating the effect of additional water resistance during the flexion/extension exercises, Aquafins (Thera-Band, Germany) were fixed at the ipsilateral ankle (Fig 1).

Furthermore, the effect of water height on the joint load was analyzed during one-legged stance. To decrease the water height 4 platforms with a height of 4 cm each were used. Due to different heights of the patients a water level at chest height was achieved with different numbers of platforms.

Verbal instructions were given to the subjects as to how to perform the activities and possibly how to correct the movement. If needed, support was provided by a floating device or the instructor. Four TKR subjects were not able to perform the dynamic activities on land, two TKR subjects did not perform the ‘very fast’ knee flexion/extension activity. Each exercise was repeated at least 6 times.

### Data analysis

For every activity the peak values of the resultant joint contact force F_res_ during the single trials were identified and averaged over the repeated trials intra-individually.

To investigate the load reducing effect of water, peak joint forces during dynamic, weight-bearing and (slow) non-weight-bearing exercises on land and in water were compared. Primarily, the joint forces were compared for each group of exercise. For each patient the median peak value of each activity group, i.e. for A: 3 non-weight-bearing exercises, B: 5 weight-bearing exercises and C: 4 dynamic exercises was determined and taken for further analysis. Differences between peak joint forces on land and in water, with n = 6, were then tested for significance in each group using a Wilcoxon test with a significance level of p = 0.05. Subsequently, joint forces on land and in water were compared for each of the 12 activities.

Furthermore, the influence of movement velocity and additional water resistance (Aquafins) on the joint loading was analyzed during hip (THR subjects) and knee flexion/extension (TKR subjects). Differences were tested for significance using a Wilcoxon test with a significance level of p = 0.05.

Pearson’s correlation coefficient was used to examine the linear relationship between water level and joint forces during one-legged stance. Descriptive statistics were used if less than 6 subjects were able to perform the exercise.

To depict the average load pattern during walking on land and in water, a dynamic time warping procedure was used [23].

## Results

### Influence of the buoyant force during one-legged stance

**Fig 2:**
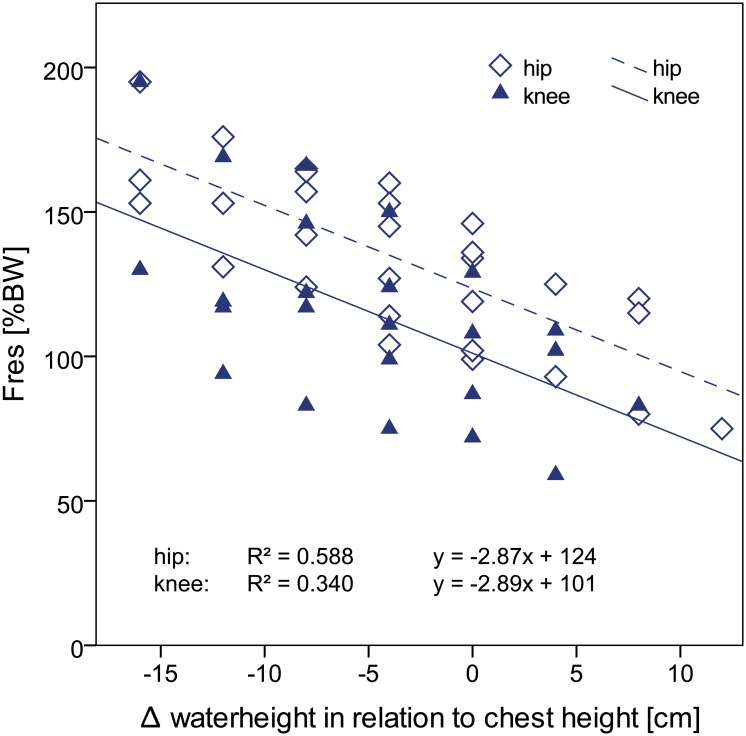
Peak resultant hip and knee joint force (F_res_) during one-legged stance in water with different water height levels. 0 cm corresponds to a chest-high water level. Due to different patient heights a water level at chest height was achieved with different numbers of platforms. BW = bodyweight.

During the static condition of one-legged stance, peak resultant hip and knee joint forces were on average reduced by 170%BW (58%) and 162%BW (62%), respectively in chest-high water when compared to one-legged stance on land. An increasing water level resulted in significantly lower hip and knee joint forces (Fig 2). Linear regression analysis between water height and joint forces revealed a significant (hip: p<0.001, knee: p = 0.002) negative correlation. With a value of 2.9 the slope of the regression line was the same for the hip and the knee joint. The y-intercept was 23%BW higher in the hip than in the knee joint.

### Hip and knee joint forces during exercises on land and in water

**Fig 3:**
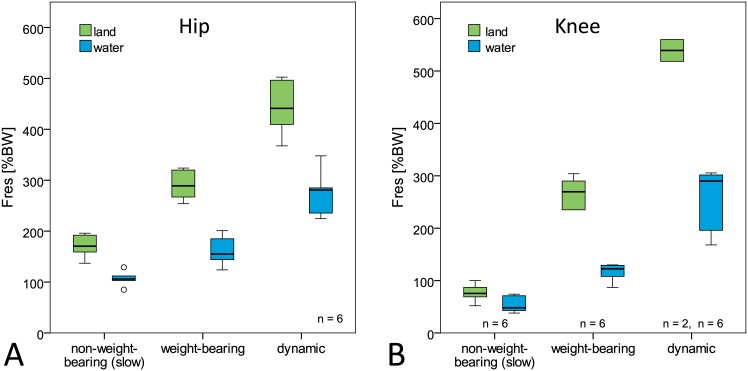
Peak hip (A) and knee (B) joint forces during exercises in water and on land. F_res_: resultant joint force. BW = bodyweight.

Resultant joint forces during the exercises on land were highest during dynamic activities (median peak values hip: 441%BW, knee: 539%BW), followed by weight-bearing (hip: 289%BW, knee: 270%BW) and slow non-weight-bearing activities (hip: 171%BW, knee: 76%BW). Compared to joint forces on land, significant force reductions were observed for all exercise groups in water (Fig 3, Table 3).

**Table 3:** Joint force reduction during exercises in water compared to land. Absolute and relative differences between median peak values on land and in water are given per group. P-values are only stated for a sample size of n = 6 per joint.

	hip		knee	
Exercise group	reduction	p-value	reduction	p-value
(A) non-weight-bearing (slow)	65%BW (38%)	0.028	28%BW (36%)	0.028
(B) weight-bearing	134%BW (46%)	0.027	147%BW (55%)	0.027
(C) dynamic	161%BW (36%)	0.028	249%BW (46%)	-

During the same activities in water the peak joint forces were reduced on average by 36–46% in the hip joint and 36–55% in the knee joint (Table 3). The highest force reduction was observed during weight-bearing and dynamic activities and exceeded 100%BW in both joints. Nevertheless, individual joint forces in water reached values of up to 396%BW (hip) and 368%BW (knee) during dynamic exercises.

**Fig 4:**
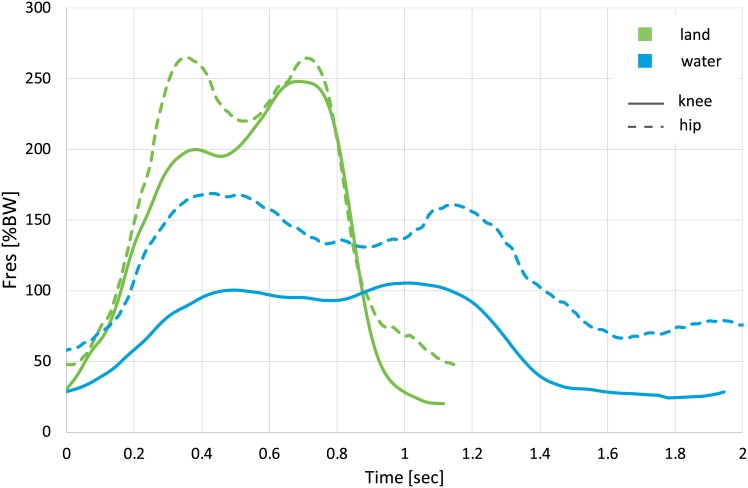
Resultant hip and knee joint force (F_res_) patterns during walking on land and in water. Average force pattern of all subjects. BW = bodyweight.

Exemplarily, the average force patterns during walking on land and in water are given in Fig 4. On land, knee and hip joint forces were of similar magnitude during the late stance phase. However, in water maximum joint forces were 39% smaller in the knee than in the hip joint. The absolute peak forces during the stance phase were reduced by 35% (hip) and 56% (knee) on average. Besides the peak forces the walking speed was reduced in water in both subject groups with the stride cycle duration in water being about twice as long as the one on land.

Hip and knee joint forces during all 12 investigated activities in water and on land can be found in the Appendix (S1–S3 Figs).

### Influence of increased water resistance on joint forces during flexion/extension

**Fig 5:**
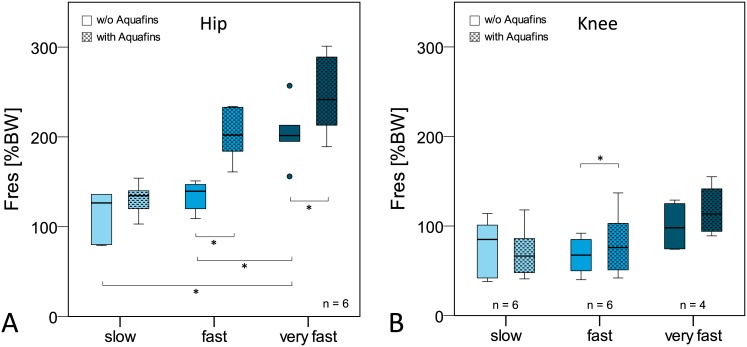
Influence of movement velocity and Aquafins on peak resultant hip (A) and knee (B) joint force (F_res_) during hip and knee flexion/extension. BW = bodyweight.

In general, increased water resistance led to an increase in peak joint force during the flexion/extension activity (Fig 5).

Hip joint: Both an increase in movement velocity and the use of Aquafins led to a significant increase of the hip joint force during *hip* flexion/extension (Tables 4 and 5). Highest hip joint forces occurred during very fast flexion/extension with Aquafins and reached up to 301%BW (Fig 5A).

**Table 4:** Influence of increased movement velocity on peak joint forces in water during flexion/extension. Absolute and relative differences between median peak forces are given (increase (+), decrease (-)). P-values are only stated for a sample size of n = 6 per joint.

	hip		knee	
acceleration	increase	p-value	increase	p-value
slow–fast	+13%BW (+10%)	0.156	-18%BW (-21%)	1.000
fast–very fast	+62%BW (+44%)	0.031	+31%BW (+45%)	-
slow–very fast	+75%BW (+59%)	0.031	+13%BW (+15%)	-

**Table 5:** Influence of increased water resistance (Aquafins) on peak joint forces during flexion/extension. Absolute and relative differences between median peak forces are given (increase (+), decrease (-)). P-values are only stated for a sample size of n = 6 per joint.

	hip		knee	
velocity	increase	p-value	increase	p-value
slow	+8%BW (+6%)	0.218	-19%BW (-22%)	1.000
fast	+63%BW (+45%)	0.031	+9%BW (+13%)	0.031
very fast	+40%BW (+20%)	0.031	+16%BW (+16%)	-

Knee joint: The effects of higher velocity and additional resistance by the Aquafins during *knee* flexion/extension on knee joint forces were less pronounced (Fig 5B). A trend towards increased knee joint loads with higher movement velocity and Aquafins was observed (Tables 4 and 5). However, only during the fast movement a significant difference was detected between the activities with and without Aquafins. Highest knee joint forces occurred during very fast movement with Aquafins and reached up to 155%BW.

### Controlled increase of peak joint forces during aquatic exercise

**Fig 6:**
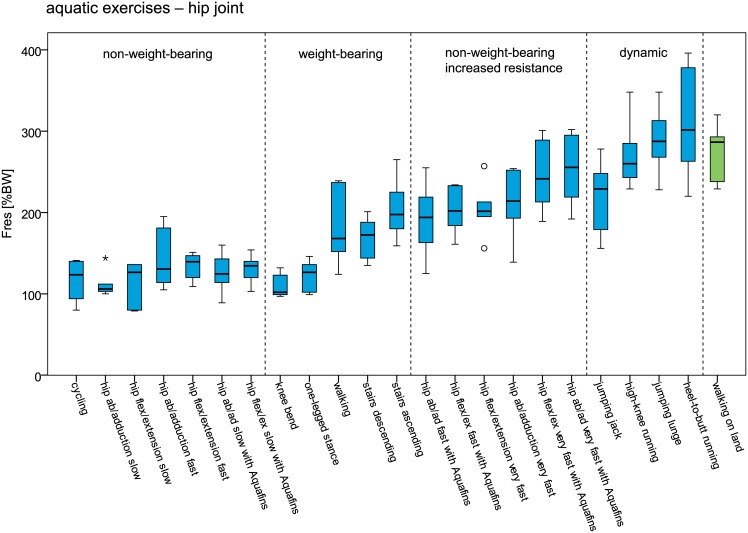
Peak resultant hip joint forces (F_res_) during aquatic exercises and walking on land. Forces are given in % bodyweight (BW).

**Fig 7:**
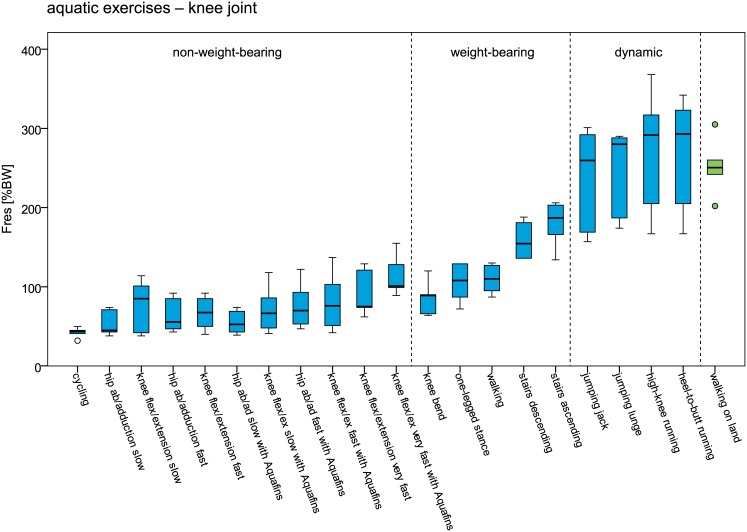
Peak resultant knee joint forces (F_res_) during aquatic exercises and walking on land. Forces are given in % bodyweight (BW).

In Figs 6 and 7 peak hip and knee joint forces during aquatic exercise in ascending order is provided and compared to walking on land. Lowest joint forces occurred generally during slow non-weight-bearing activities, followed by weight-bearing activities and dynamic activities. During dynamic aquatic exercises peak hip and knee joint forces were in a similar range as during walking on land. However, also during non-weight-bearing hip flexion/extension and ab-/adduction with increased water resistance, peak hip joint forces reached a similar magnitude.

Selected datasets of all investigated exercises including data and video files can be found in the ‘Orthoload’ database (www.orthoload.com).

## Discussion

Aquatic exercises are frequently recommended for rehabilitation or preventive therapies in order to enable mobilization and muscle strengthening while minimizing the joint loads of the lower limb. However, so far reliable data about the effect of aquatic exercise on the joint loading was lacking. This study is the first one reporting *in vivo* measured joint forces during aquatic exercises and demonstrates the load reducing effect of water compared to exercises on land.

In order to understand the effect of water on joint loads the water properties have to be recognized. The buoyant force counteracts the gravitational force and leads to a reduction of the apparent body weight F_a_. In chest-high water F_a_ decreases by about 65–68% to only 32–35% body weight [24]. The results of this study confirm the load reducing effect of water in the static condition. Similar to F_a_, the joint force was reduced by 58% (hip) and 62% (knee) during one-legged stance in chest-high water. In absolute values, joint forces were reduced by more than 100%BW which indicates that not only the apparent body weight but also muscle forces were reduced. Every 10 cm decrease/increase of the water level led to an increase/decrease of the joint forces by 29%BW.

In the dynamic situation however, not only buoyancy but also drag forces that act opposite to the relative motion of the body segments have to be considered. An increase of drag forces due to a higher movement velocity or larger surface area has been demonstrated during knee flexion/extension using a human leg/foot model [25]. Drag forces lay in a range of 20–60N, depending on movement velocity, and increased about tenfold with a 30% increased surface area. To overcome those drag forces higher muscular activity is required which in turn may lead to increased joint forces. The results of this study show that both, a higher movement velocity as well as an increased water resistance due to a higher surface area lead to increased joint forces. This finding was more pronounced for the hip than the knee joint which can probably be explained by the differences in exercise and surface area counteracting the drag forces. Whereas the THR subjects performed a hip flexion/extension activity with a surface area consisting of the cross sections of thigh and shank, TKR subjects performed a knee flexion/extension activity with only the shank as surface area. Hip joint forces of up to 301%BW during the very fast hip flexion/extension clearly demonstrate the great effect of muscle forces on the joint loading. Furthermore, it indicates that aquatic exercises can be used effectively for muscle activation.

In order to restore the patients’ competence in performing daily tasks a controlled increase of joint loads and range of motion is often aimed for throughout a rehabilitation program. The maximum joint forces during aquatic exercises lay in a wide range of 32 to 396%BW. As shown by the results, the peak joint forces can be easily modulated by the type of exercise, movement velocity and additional resistive devices. Lowest joint forces can generally be expected during slow non-weight-bearing activities, followed by weight-bearing activities and dynamic activities where peak joint forces similar to the ones during daily activities [26–28] were reached. With median peak values of 281%BW (hip) and 290%BW (knee) the joint loads during dynamic aquatic exercises were in a similar range as during walking on land. A controlled force increase can furthermore be achieved by higher movement velocities and additional resistive devices.

Furthermore, aquatic exercise aims at restoring or improving the overall mobility. Even in this small group of subjects it became apparent that certain dynamic exercises could not be performed on land by some subjects whereas in the water the same exercise was easily accomplished.

A limitation of this study is the small sample size of only 6 patients per group and multiple comparisons were performed leading to an increased risk of false positive results. Due to the small sample size only prominent effects can be detected with this study design and the exploratory rather than confirmatory character of this study has to be acknowledged. However, this study presents the greatest group of patients with instrumented implants currently available and is the first one reporting *in vivo* measured joint forces in water.

Furthermore, a limitation of this study is the fact that the movement velocity, which has an influence on joint loading, was only controlled for during the non-weight-bearing activities. In general, the self-chosen movement velocity during weight-bearing and dynamic exercises was slower in water than on land. However, this study aims at addressing the clinical practice where the movement velocity in water is generally considerably slower that on land.

In conclusion, the results of this study confirm the load reducing effect of water during weight-bearing and dynamic exercises. Nevertheless, high drag forces result in increased joint contact forces and indicate greater muscle activity. By the choice of activity, movement velocity and additional devices the joint forces can be modulated individually in the course of rehabilitation or preventive therapies.

## Supporting information

S1 Fig Peak resultant hip and knee joint forces (F_res_) during non-weight-bearing activities. BW = bodyweight. *p<0.05, Wilcoxon test. P-values are only stated for a sample size of n = 6 per joint.
(EPS)

S2 Fig Peak resultant hip and knee joint forces (F_res_) during weight-bearing activities. BW = bodyweight. *p<0.05, Wilcoxon test.
(EPS)

S3 Fig Peak resultant hip and knee joint forces (F_res_) during dynamic activities. BW = bodyweight. *p<0.05, Wilcoxon test. P-values are only stated for a sample size of n = 6 per joint.
(EPS)
